# Clinico-Pathological Importance of TGF-β/Phospho-Smad Signaling during Human Hepatic Fibrocarcinogenesis

**DOI:** 10.3390/cancers10060183

**Published:** 2018-06-05

**Authors:** Katsunori Yoshida, Koichi Matsuzaki, Miki Murata, Takashi Yamaguchi, Kanehiko Suwa, Kazuichi Okazaki

**Affiliations:** Department of Gastroenterology and Hepatology, Kansai Medical University 2-5-1, Shin-Machi, Hirakata, Osaka 573-1010, Japan; matsuzak@takii.kmu.ac.jp (K.M.); muratami@takii.kmu.ac.jp (M.M.); yamaguct@hirakata.kmu.ac.jp (T.Y.); suwakan@hirakata.kmu.ac.jp (K.S.); okazaki@hirakata.kmu.ac.jp (K.O.)

**Keywords:** transforming growth factor (TGF)-β, Smad, hepatic stellate cells (HSC), myofibroblasts (MFB), liver fibrocarcinogenesis

## Abstract

Chronic viral hepatitis is a global public health problem, with approximately 570 million persons chronically infected. Hepatitis B and C viruses increase the risk of morbidity and mortality from liver cirrhosis, hepatocellular carcinoma (HCC), and extrahepatic complications that develop. Hepatitis virus infection induces transforming growth factor (TGF)-β, which influences microenvironments within the infected liver. TGF-β promotes liver fibrosis by up-regulating extracellular matrix production by hepatic stellate cells. TGF-β is also up-regulated in patients with HCC, in whom it contributes importantly to bringing about a favorable microenvironment for tumor growth. Thus, TGF-β is thought to be a major factor regulating liver fibrosis and carcinogenesis. Since TGF-β carries out regulatory signaling by influencing the phosphorylation of Smads, we have generated several kinds of phospho-specific antibodies to Smad2/3. Using these, we have identified three types of phospohorylated forms: COOH-terminally phosphorylated Smad2/3 (pSmad2C and pSmad3C), linker phosphorylated Smad2/3 (pSmad2L and pSmad3L), and dually phosphorylated Smad3 (pSmad2L/C and pSmad3L/C). TGF-β-mediated pSmad2/3C signaling terminates cell proliferation; on the other hand, cytokine-induced pSmad3L signaling accelerates cell proliferation and promotes fibrogenesis. This review addresses TGF-β/Smad signal transduction in chronic liver injuries and carcinogenic processes. We also discuss the reversibility of Smad signaling after antiviral therapy.

## 1. Introduction

Hepatitis B and C virus (HBV and HCV) infection are major global public health problems. Worldwide, HBV is estimated to have infected over two billion persons; more than 400 million of them are considered chronically infected [1]. Some 2% to 3% of the world’s population, up to 170 million individuals, are believed to be infected with HCV [1]. Virally-associated ongoing liver inflammation can cause liver fibrosis and damage DNA in regenerative hepatocytes. Fibrosis and inflammation significantly increase the likelihood of genetic alterations that promote hepatocellular carcinoma (HCC) development, which occurs at a rate of around 7% per year in persons with HCV-related cirrhosis [2]. On the other hand, HCC occurs at 3.7% per year among patients with HBV-related cirrhosis [3].

Many growth factors and cytokines regulate extracellular matrix (ECM) production. Transforming growth factor (TGF)-β is the most potent of these in accelerating liver fibrosis [4,5]. TGF-β enhances the activation of hepatic stellate cells (HSC), stimulates collagen gene transcription, and suppresses matrix metalloproteinase (MMP) expression. Thus, TGF-β signaling should represent a potential therapeutic target for treating liver fibrosis. Additionally, the TGF-β signaling pathway can interfere with hepatocyte proliferation, but it also can accelerate HCC progression. TGF-β has been reported to play both tumor-suppressive and tumor-promoting roles [6,7,8]. As disease progresses toward malignancy, HCC selectively reduces the tumor-suppressive activity and augments the oncogenic activity of TGF-β [7]. In cooperation with mitogens, TGF-β promotes ECM deposition, while mitogenic signaling antagonizes cytostatic TGF-β function [9,10,11]. Recently, the possibility of Smads family involvement in the pathogenesis of fibrosis and carcinogenesis (fibrocarcinogenesis) has been emphasized. 

Recent studies have proven that Smads are tightly controlled as TGF-β signaling mediators by domain-specific phosphorylation, which regulates subcellular localization, transcriptional response, and the stability of their components [12,13,14]. Accordingly, monitoring the phosphorylation status of signaling molecules plays a key part in dissecting their pathways. In this review, we describe how phospho-Smads transmit cell type-specific and context-dependent signals. We next summarize TGF-β signaling during human chronic hepatitis. We then discuss reversibility of phospho-Smad signaling after antiviral therapy against chronic hepatitis. We finally consider prevention and molecular targeting therapy for HCC.

## 2. Multiple Smad Phospho-Isoforms Signaling Exist

**Figure 1:**
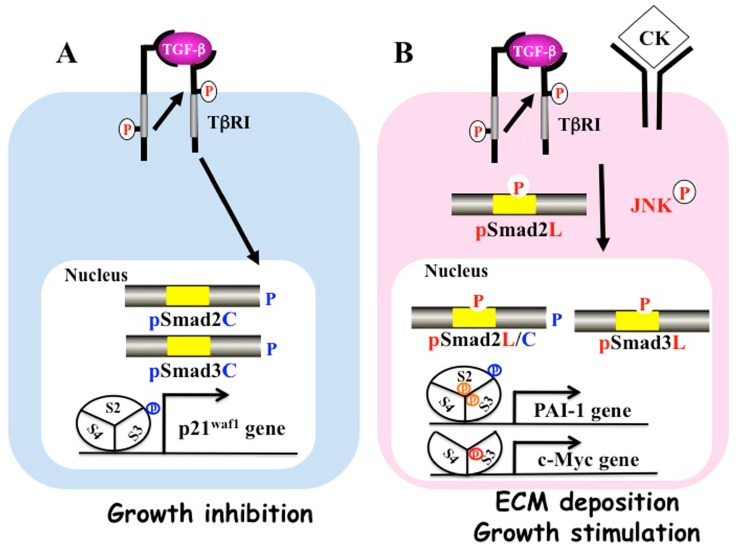
Differential phospho-Smad signals between tumor suppression and fibrocarcinogenesis. (**A**) Activated transforming growth factor (TGF)-β type I receptor (TβRI) phosphorylates COOH-tail serine residues of Smad2 and Smad3. Both COOH-terminally phosphorylated Smad2/3 (pSmad2C and pSmad3C) translocate with Smad4 to the nuclei of quiescent hepatocytes after regeneration. Smad2/3/4, complex binds the p21^waf1^ promoter and suppresses cell growth; (**B**) Pro-inflammatory cytokines (CK) such as tumor necrosis factor-α activate c-Jun N-terminal kinase (JNK), which phosphorylates the linker regions of Smad2 and Smad3. Linker phosphorylated Smad3 (PSmad3L) translocates with Smad4 to the nucleus and binds plasminogen activator inhibitor type 1 (PAI-1) promoter. Linker phosphorylated Smad2 (PSmad2L) is localized in the cytoplasm, and Smad2 translocates to the nucleus only after COOH-tail phosphorylation by TβRI. PSmad2L/C in cooperation with pSmad3L and Smad4 stimulate PAI-1 transcription and extracellular matrix (ECM) deposition. PSmad3L up-regulates c-Myc and stimulates cell growth, while suppressing the pSmad3C-mediated tumor suppressive pathway.

TGF-β binds to the type II receptor on the cell surface, recruiting the TGF-β type I receptor (TβRI). Activated TβRI phosphorylates the COOH-terminal regions of Smad2 and Smad3. After forming hetero-oligomers with Smad4, they translocate from the cytoplasm to the nucleus, where they regulate gene transcription [13] (Figure 1A). This pathway is regulated by several auto-inhibitory feedback loops. Smad7, which is expressed in response to prolonged TGF-β signal, inhibits TGF-β signaling [15,16].

Although C-terminal SXS phosphorylation by the TβRI is the key event in Smad activation, phosphorylation by intracellular protein kinases also positively and negatively regulates Smads. Smad2 and Smad3 consist of a conserved MH1 domain that binds DNA, and a conserved MH2 domain that binds receptors, a partner Smad4, and transcriptional co-activators [13]. More divergent linker regions separate the two domains. There are one ThrPro (TP) and three SerPro (SP) cluster sites in Smad2/3 linker regions. First, the TP site of Smad2 (Thr-220) and Smad3 (Thr-179) are almost the same. Three SP sites of Smad2 (Ser-245, Ser-250, and Ser-255) are different from that of Smad3 (Ser-204, Ser-208, and Ser-213). Sequences around Smad2 (Ser-255) and Smad3 (Seer-213) especially differ from each other [17]. The linker domains are phosphorylated by both cytoplasmic mitogen-activated protein kinase (MAPK), and members of the nuclear cyclin-dependent kinase (CDK) family [9,14,17,18,19,20]. MAPKs including extracellular signal-regulated kinase (ERK) 1/2, c-Jun N-terminal kinase (JNK)1/2/3, and p38/MAPKs, regulate a variety of cellular events.

The linker regions of Smad2/3 contain serine/threonine residues, and individual sites are phosphorylated by specific kinases. For instance, TGF-β phosphorylates more often at Thr-220/179 in Smad2/3 [21,22,23,24,25]. On the other hand, receptor tyrosine kinase (RTK) growth factors such as the epidermal growth factor, hepatocyte growth factor, and platelet-derived growth factor, as well as pro-inflammatory cytokines such as tumor necrosis factor (TNF)-α and interleukin-1β, phosphorylate 3SP sites much more strongly than at Thr-220/179 [26,27,28,29]. These data suggest that the linker region of Smads plays an important role in the cross-talk between TGF-β and other cytokines [9,14,20].

Since antibodies (Abs) specific for each phosphorylation site of linker regions are indispensable reagents for detailed analysis, we immunized phosphorylated peptides and obtained several kinds of domain-specific phospho-Smad Abs. These Abs were useful for the assessment of individual pThr/pSer residues in the linker segments of Smad2 and Smad3 [19,20,23]. Moreover, we have revealed the mechanisms how these sites are phosphorylated by JNK, TβRI, and CDK. Finally, we concluded that there are three types of phospho-isoforms: C-terminally phosphorylated Smad2/3 (pSmad2C and pSmad3C), linker-phosphorylated Smad2/3 (pSmad2L and pSmad3L), and dually phosphorylated Smad2/3 (pSmad2L/C and pSmad3L/C) [9]. As Smad activity can be related to different genetic and epigenetic backgrounds of different cellular systems that one might analyze such as normal cells versus immortalized cells and cancer cells, differences or apparent contradictions are likely between various studies concerning Smad linker phosphorylation and its consequences. 

## 3. PSmad3C Displays Cytostatic Activity

In normal epithelial cells, TGF-β halts cell proliferation. CDK, cyclins, and CDK inhibitors play important roles in both TGF-β and Ras signaling [19,21]. Growth inhibition by TGF-β occurs via interference with cell cycle progression. TGF-β-dependent pSmad3C signaling interferes with cell-cycle progression at the early to mid G_1_ phase by the transcriptional activation of p15^INK4B^ and p21^CIP1^ and the repression of c-Myc genes [30,31,32]. Cancer development is impeded by the pSmad3C signaling pathway, which can cause normal epithelial cells to cease growth and enter apoptosis after transient Ras activation, partly through the ability of pSmad3C to induce or repress the expression of a number of apoptosis-associated proteins such as Bcl2 [33]. 

## 4. PSmad3L (Ser-213) Enhances Mitogenic Pathways

JNK, a serine/threonine kinase, promotes cell proliferation, differentiation, survival, and migration [34,35]. We have focused on the linker phosphorylation of Smad3 at Ser-213 induced by the Ras/JNK pathway [23]. Although pSmad2L (Ser-245/250/255) and pSmad3L (Ser-204) remain in the cytoplasm, pSmad3L (Ser-213) resides in the nucleus, permitting further consequences of JNK signaling. Smad3L at Ser-213 is phosphorylated by RTK growth factors, pro-inflammatory cytokines, and to a lesser extent, TGF-β. Trimers including two Smad2/3s and one Smad4 are thought to act as the critical functional units [36,37]. In the nucleus, Smad2/3 and Smad4 make complexes with other DNA-binding transcription factors and regulate transcription. Both pSmad3C and pSmad3L (Ser-213) make hetero-complexes with Smad4, after which the Smad complex translocates to the nucleus [26]. Since hetero-oligomerization is needed to bind target-specific transcriptional complexes in the nucleus [38], two different types of phospho-Smad3 transmit different signals, which is consistent with the ability of Smad4 to act as both a tumor repressor and a tumor activator [39]. Additionally, pSmad3L (Ser-213) can access multiple 5′-AGAC-3′ sequences, which are named Smad-binding elements, in the promoters of certain target genes [40]. 

In order to overcome TβRI/pSmad3C-mediated growth arrest, Ras-mediated JNK activation is essential for transformation. c-Myc overexpression has been shown to inhibit the Smad3-dependent transcription of p15^INK4B^ and p21^WAF1^, overriding cell-cycle blockade [41]. Resistance to TβRI/pSmad3C/p21^WAF1^-mediated growth arrest has been ascribed to a mitogenic pathway involving JNK/c-Myc [42]. Ser-213 phosphorylation of Smad3L interferes C-tail phosphorylation by TβRI [10,23,43]. Mitogenic signaling accelerates the nuclear translocation of pSmad3L (Ser-213) from the cytoplasm, while decreasing Smad3C phosphorylation, pSmad3C-mediated transcription, and the anti-proliferative effects of TGF-β [23]. In this manner, TβRI-induced pSmad3C-mediated cell-cycle-arrest signaling and the JNK-mediated cell-proliferation signaling antagonize each other. Since the pSmad3C pathway is also required for the maintenance of genomic stability and the induction of replicative senescence [44,45], insensitivity to pSmad3C conveyed by constitutive pSmad3L (Ser-213) signaling results in uncontrolled cell proliferation, contributing to carcinogenesis. 

## 5. PSmad2L/C and pSmad3L/C Promote Pro-Tumorigenic/Fibrogenic Pathways

In 1983, Roberts et al. reported the isolation of two fractions from murine sarcoma cell extracts that could synergistically induce remarkable growth of mesenchymal fibroblasts on soft agar, which is a hallmark of cellular transformation [46]. In that study, they found that TGF-β strongly induced transformation in normal fibroblasts, but only in the presence of TGF-α, which is a ligand for RTK that transmits mitogenic signals via the Ras pathway. This was the first important example of functional interaction between TGF-β and other mitogenic signaling pathways [14]. 

Although JNK negatively regulates pSmad3C activity in normal epithelial cells by up-regulating pSmad3L (Ser-213), JNK also synergistically activates Smad2C activity in mesenchymal cells [47]. This functional difference between Smad2 and Smad3 pathways could result from variation in amino acid sequences near the 3SP cluster in linker regions. Constitutive Ras-activation caused by mutations increases mitogenic pSmad3L (Ser-213), while decreasing TGF-β-dependent cytostatic pSmad3C function [23]. Ras mutations concurrently promote pro-tumorigenic TGF-β signaling and activate invasive behavior by the up-regulation of epithelial-mesenchymal transition (EMT)-like proteins through the pSmad2L (Ser 245/250/255)/C pathway [24]. 

Cyclin overexpression reduces cell-cycle control and promotes the potential for cellular transformation and primary tumor growth [48,49]. Cyclin D knockout mice are protected from Ras-induced cancers [50]. Moreover, Ras-transformed cells frequently amplify or overexpress the cylin D1 gene and exhibit high CDK4 activities [51]. Furthermore, cyclin D1 overexpression has been shown to promote disease progression and metastasis [48]. Mitogens and hyperactive Ras favor in the CDK-mediated phosphorylation of Smad3 at Thr-179 [22,24,25], and of Smad2 at Thr-220 [22,24,48]. The CDK-dependent phosphorylation of Smad3 interferes with the anti-proliferative action of TGF-β and serves as a novel way by which CDKs positively regulate aberrant cell cycle progression [24,52,53]. In knockdown cell lines, Smad linker phosphorylation promoted cell invasion and migration by inducing MMP 2/9 and plasminogen activator inhibitor type 1 (PAI-1) [24]. These data provide additional evidence for a switching of phospho-Smad pathways from tumor suppression to metastasis promotion. 

Activated JNK retains Smad2 proteins in the cytoplasm, while C-terminal phosphorylation of Smad2 by the TβR1 receptor is essential for accumulation in the nucleus under conditions of sustained linker phosphorylation by JNK (Figure 1B). The translocation of pSmad2L/C to the nucleus enhances PAI-1 transcription in cooperation with pSmad3L and Smad4. Smad7 has been reported to inhibit pSmad2C-mediated signaling and reduce TGF-β-mediated hepatic fibrogenesis [54]. Moreover, interferon (IFN) up-regulates Smad7 and exerts anti-fibrotic effects [55]. We also found that pSmad2L (Ser-245/250/255)/C stimulates PAI-1 expression by acting together with pSmad3L (Ser-213) to transmit fibrogenic signals. Thus, TGF-β and pro-inflammatory cytokines additively up-regulate pSmad2L/C and pSmad3L signaling, and increase PAI-1 transcription and ECM synthesis in hepatocytes and myofibroblasts (MFB), promoting liver fibrosis.

## 6. A Shift of Hepatocytic Phospho-Smad Signaling from the Tumor-Suppressive pSmad3C Pathway to Carcinogenic pSmad3L and Fibrogenic pSmad2L/C Pathways Observed during Hepatic Fibrocarcinogenesis

In the healthy state, TGF-β produced by nonparenchymal liver cells, including sinusoidal endothelial cells, Kupffer cells, and hepatic stellate cells (HSC), suppresses the proliferation of normal hepatocytes [56,57,58]. However, plasma TGF-β often is elevated in chronic hepatitis and HCC patients [59,60]. Interestingly, low mutation frequencies in TGF-β receptors and Smad proteins, which are often found to be mutationally inactivated in other gastrointestinal cancers, has been reported in transformed hepatocytes and HCC cells [61,62,63,64,65]. Thus, while TGF-β signaling is tumor-suppressive in various tissues, transformed hepatocytes and HCC cells often retain sensitivity to TGF-β.

Many clinical observations suggest that persistent hepatitis virus infection and chronic inflammation additively favor the development of HCC. HBV contains partially double-stranded DNA, which integrates into the host genome where it can directly cause HCC. The integration of HBV DNA has been observed in over 85% to 90% of livers during and sometimes even before the development of HBV-related HCC [66]. HBV genomic integration is not restricted to HCC, but it is also is found in the non-tumor tissue in patients with chronically HBV infection [67,68]. HBV integration influences a wide range of genetic alterations within the host genome, including chromosomal deletions, translocations, the production of fusion transcripts, the amplification of cellular DNA, and generalized genomic instability [69,70]. Among the possible consequences of HBV genomic integration, the HBx protein has been suspected as a viral oncoprotein promoting liver carcinogenesis. Using transgenic mice, HBx was proven to participate in c-Myc-induced hepatocarcinogenesis [71].

In HBx transgenic models, we have reported that HBx participates importantly in hepatocarcinogenesis via the pSmad3L/c-Myc pathway [43]. HBx transgenic mice have been shown to progress through hyperplasia to HCC in the liver [72]. As HBx mice underwent this progression through hyperplasia to HCC, hepatocytic HBx, pSmad3L, and c-Myc increased. The phosphorylation of Smad3L in the nuclei of hepatocytes increases in step with the amount of HBV DNA in early chronic hepatitis B specimens [43]. These data suggest that that the HBx oncoprotein directly participates in hepatocarcinogenesis by shifting hepatocytic phospho-Smad3 signaling from the tumor-suppressive pSmad3C/p21^WAF1^ pathway to the oncogenic JNK/pSmad3L/c-Myc pathway [43]. 

Since HCV is a positive single-strand RNA virus, it cannot integrate into the host’s genome. Instead, HCV components modulate many cellular regulatory functions by targeting a broad spectrum of cell signaling systems [73,74,75,76,77,78,79,80]. The HCV core protein has been shown to activate the JNK pathway and regulate the vascular endothelial growth factor [80]. Nonstructural protein 5A (NS5A) also activates the JNK signaling pathway by interacting with TNF receptor-associated factor 2, which may be extremely important in HCV-related liver disease [81]. Lin et al. have shown that HCV can directly induce TGF-β release from hepatocytes in a reactive oxygen species (ROS)-dependent and a JNK-dependent manner in an HCV infection model [82]. Moreover, recent studies demonstrated that HCV is directly involved in hepatocarcinogenesis in transgenic mouse models. Liver steatosis and HCC have been observed in three different HCV core transgenic lines [83,84,85]. 

**Figure 2:**
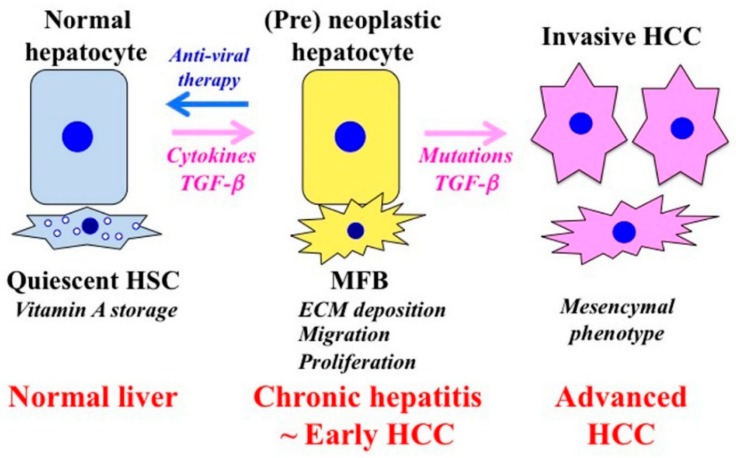
Phenotypic alternations of hepatocytes and HSC during the fibrocarcinogenic process in human chronic liver diseases. Quiescent hepatic stellate cells (HSC) are characterized by retinoid droplets in the cytoplasm and maintain liver homeostasis. HSC undergo constitutive activation to become myofibroblasts (MFB)-like cells after liver injury. MFB persistently produce an extracellular matrix (ECM) and induce liver fibrosis. The contraction of MFB contributes to increased portal resistance during liver fibrosis that presumably is reversible until the thickened septae, intrahepatic shunts, and lobular distortion that are characteristic of cirrhosis development, leading to fixed increases in portal pressure. Chronic liver damage promotes recurrent cycles of cellular proliferation, inflammation, fibrosis, and carcinogenesis. In pre-neoplastic hepatocytes, several growth factors and cytokines activate proliferation and invasion. As human hepatitis virus-related chronic liver diseases progress, chronic inflammation and hepatitis virus additively accelerate liver fibrosis and increase the risk of hepatocellular carcinoma (HCC). Genetic and epigenetic changes in the liver result in carcinogenesis. Effective antiviral therapy can reverse the pre-neoplastic properties of hepatocytes to a tumor-suppressive mode before the occurrence of genetic mutations that have been implicated for HCC occurrence.

Liver fibrosis also promotes carcinogenesis. Activated HSC secretes large amounts of ECM proteins after liver injury. Hepatocytes are surrounded by abundant ECM, mainly in the form of fibrillar collagen. Affected hepatocytes also stimulate the deposition of ECM proteins and participate in liver fibrogenesis. Several soluble factors, including growth factors, cytokines, chemokines, and oxidative stress products take part in the activation of HSC and hepatocytes. In the presence of chronic liver tissue damage and inflammation, these factors are simultaneously active in the tissue and are partly, perhaps largely, responsible for the fibrocarcinogenic process. Tissue environment plays an essential role for tumor formation and development [86]. Carcinogenesis involves the transition of a normal cell into a pre-neoplastic lesion that develops into a malignant tumor [87]. Chronic liver inflammation promotes HSC activation to become MFB, which produce components of the ECM that promote fibrosis. This process is associated with the distortion of the parenchyma, which is characterized by the deposition of basement membrane components within the space of Disse. The interaction of different cell types in the ECM results in the acquisition of an abnormal phenotype that causes transformation. The stromal components support tumor growth and promote invasion through the stimulation of hepatocyte proliferation, migration, and invasion, which together promote the transformation of normal hepatocytes into pre-neoplastic hepatocytes (Figure 2).

In patients with chronic liver disease progression from chronic hepatitis to liver cirrhosis and HCC, we have demonstrated that pro-inflammatory cytokine and viral components drive carcinogenesis by shifting the tumor-suppressive pSmad3C pathway to the oncogenic pSmad3L pathway. This signaling change is also observed during the transdifferentiaton of HSC to MFB [10]. Affected hepatocytes cooperatively promote liver fibrosis by stimulating the deposition of ECM proteins. As shown in MFB, hepatocytes in chronically injured livers, particularly those adjacent to inflamed portal tracts, exhibit Ser-213 phosphorylation at Smad3L [27]. Thus, hepatocytes are also regulated by the same pSmad3L (Ser-213) pathway. The extent of phosphorylation at Smad3L (Ser-213) is less in hepatocytes that are distant from portal tracts; this is in sharp contrast to pSmad3C, which is located predominantly in hepatocytic nuclei distant from portal tracts [27]. Infiltrating Kupffer cells in portal tracts secrete TGF-β and pro-inflammatory cytokines to activate JNK [88,89]. These finding demonstrate that pro-inflammatory cytokine-dependent JNK converts Smad3 to pSmad3L (Ser-213) in both affected hepatocytes and MFB in chronic hepatitis.

Clinical research has supported our experimental data. The strong phosphorylation of Smad3L is observed in HCC specimens as well as in human HCC cell lines [90]. Interestingly, specimens from chronic hepatitis B patients who develop HCC show high levels of linker Smad3 phosphorylation, but low levels of C-terminal Smad3 phosphorylation in hepatocytic nuclei, whereas other patients with a high level of hepatocytic pSmad3C but a low level of pSmad3L do not develop HCC [43]. Similar patterns are observed in patients with hepatitis C virus-related HCC [27]. Taken together, HBV or HCV components and pro-inflammatory cytokine additively activate JNK to shift Smad phospho-isoform signaling from the tumor-suppressive TβRI/pSmad3C pathway to the carcinogenic JNK/pSmad3L pathway together with the fibrogenic pSmad2L/C pathway, accelerating liver fibrosis and promoting hepatocarcinogenesis.

## 7. Effective Antiviral Treatment Can Reverse Phospho-Smad Signaling in Early Stages of Chronic Liver Disease

IFN-based therapy can achieve approximately 50% to 60% rates of sustained virological response (SVR) rates [91]. The next advance was asunaprevir and daclatasvir, representing the first fully orally administered, IFN-free direct-acting antivirals (DAA) approved for treating patients with HCV genotype 1 infections [92]. Sofosbuvir/ledipasvir and paritaprevir/ritonavir/ombitasvir demonstrate approximately 95% SVR rates [93,94]. More recently, glecaprevir/pibrentasvir, which shows high SVR rates against every genotype of HCV, has been approved [95]. Likewise, nucleoside analogues are recognized as being more effective for chronic hepatitis B patients than IFN therapy. Five nucleoside analogues have been licensed: lamivudine [96]: adefovir (in 2002) [97], entecavir (in 2005) [98], telbivudine (in 2006) [99], and most recently, tenofovir (in 2008) [100]. These nucleoside analogues inhibit reverse transcriptase and DNA polymerase suppressing HBV replication. They also inhibit the reverse transcription of pregenomic RNA to HBV DNA [96]. 

Previous studies have shown that successful antiviral therapy can improve both biochemical liver function parameters and liver histology [101]. Suzuki et al. had classified the degree of liver fibrosis from stage F0 (no fibrosis) to stage F4 (cirrhosis) by Desmet’s histological classification and reported a −0.6 fiborosis regression score in the liver specimens from chronic hepatitis B patients after one year of anti-HBV therapy [102]. We have reported that fibrosis improved by −1 point after one year of anti-HBV treatment [103]. In chronic HCV infection, the fibrosis regression rate was only −0.28 point per year after SVR [104]. A comparison of results indicates that anti-HBV therapy using nucleoside analogues resulted in three to four times more rapid hepatic fibrosis regression in HBV than that could be achieved in IFN-treated HCV. Notably, fibrocarcinogenesis regression with treatment was much faster in HBV than in HCV-related liver disease [103]. These data are compatible with clinical observations that after oral nucleoside therapies, ascites or jaundice often disappear in decompensated HBV-infected patients, even those in advanced stages. Since hepatic fibrocarcinogenesis is a very complex process, differences in virus-specific genetic changes and their biologic consequences might cause disparities in fibrosis regression rates between HBV and HCV. Such differences might influence reversibility with antiviral therapy of early carcinogenesis as well. 

Liver histology was effectively improved in early-stage HCV patients after HCV treatment [101]. Moreover, SVR reduces the risks of decompensated liver disease and HCC occurrence [2,105]. However, patients with advanced fibrosis retain relatively low but still considerable risks of HCC occurrence and hepatic decompensation, despite having attained SVR [105]. Our recent data are consistent with such guarded optimism. When we analyzed pSmad3L and pSmad3C before and after, antiviral therapy, treatment could shift Smad phospho-isoform signaling from the oncogenic pSmad3L pathway to the normal pSmad3C pathway in early stages of either chronic hepatitis B or C [103,104]. The clearance of hepatitis virus decreased pro-inflammatory cytokines in the liver, with the consequent down-regulation of the linker-region phosphorylation of Smad3. In contrast, patients with advanced liver fibrosis progressed to HCC despite decreased inflammatory activity with treatment. In these patients, hepatocytes maintained high levels of Smad3L phosphorylation and low levels of Smad3C phosphorylation [104]. These data suggest that if genetic and epigenetic changes have already been obtained, liver inflammation no longer plays critical roles in HCC occurrence in the late stages of cirrhotic livers. These data also prompt us to perform prospective study as to whether pSmad3L can become a useful marker for liver fibrosis and HCC incidence. We speculate that a group that keeps high pSmad3L levels after antiviral therapy still has a high risk for hepatic decompensation and HCC risk, and that in a group in which pSmad3L levels improved decreased liver fibrosis and risks of HCC. We are performing a detailed analysis of TGF-β signaling in paired biopsy specimens after successful antiviral therapy.

## 8. Implications for HCC Prevention and Therapy

Both fibrosis and HCC incidence is impaired in JNK^−/−^ mice. Liver fibrosis is less dense in JNK1^−/−^ mice compared with that of wild-type and JNK2^−/−^ mice [106]. Moreover, HCC incidence was rare and the size of HCC was smaller in JNK1^−/−^ mice [42]. These data suggest that JNK1 is essential for liver fibrocarcinogenesis. JNK1^−/−^ mice have been demonstrated to increase p21^WAF1^ expression and reduce c-Myc expression, and thereafter decrease HCC in a carcinogenic model. On the other hand, hepatocyte proliferation was inhibited in liver regeneration models by the same mechanisms.

JNK phosphorylates the linker region of Smad3, which in turn increases cell growth and interferes with cytostatic pSmad3C signaling. Conversely, inactivating JNK-mediated carcinogenic pSmad3L signaling can restore the tumor-suppresive pSmad3C pathway and stop carcinogenesis [107]. The JNK inhibitor has been proved to inhibit HCC incidence by decreasing pSmad3L and restoring pSmad3C [108].

Further research, including studies of small molecules inhibiting JNK and pSmad3L pathways, is necessary for improved molecular targeting of HCC therapy that might substantially improve prognosis.

## 9. EMT in Liver Fibrosis and HCC Progression

EMT and its opposite, mesenchymal-epithelial transition (MET), have been reported to be important for liver fibrocarcinogenesis [109]. The process of EMT allows a polarized epithelial cell to undergo multiple biochemical changes to convert into a mesenchymal cell phenotype [110]. When EMT occurs in a normal epithelial cell, these cells lose cell polarity and get migration ability [109]. An EMT also increases ECM deposition and anti-apoptosis properties. Based upon biologic context, three types of EMT have been classified. Type 1 EMT promotes organ development. Type 2 EMT is closely related to organ regeneration and fibrosis. Type 3 EMT is associated with cancer invasion and metastasis [111]. TGF-β plays essential roles in initiation and the completion of EMT [112].

Type 2 EMT is observed during tissue repair, in which epithelial or endothelial cells transition to become resident tissue fibroblasts in response to chronic inflammation. TGF-β has been shown to decrease epithelial and hepatic markers, such as E-cadherin and albumin, and upregulate mesenchymal markers, such as vimentin, α-smooth muscle actin (α-SMA), and β-catenin [66,113,114,115,116]. Following a liver injury of any etiology, HSC undergo activation. Injured Kupffer cells and endothelial cells constitutively produce many kinds of cytokines, which can change activated HSC to MFB [117]. TGF**-**β, platelet derived growth factor (PDGF), and endothelin**-**1 can regulate gene expression by activating several kinds of signal transducer, such as Sp1, c**-**Jun, STAT**-**1, and Smad [118,119,120,121]. In order to maintain an activated status, MFB constitutively produce TGF-β and express its receptor on the cell surface without down-regulation [122]. Cells expressing α-SMA and vimentin accumulate in fibrotic septa [123,124]. Interestingly, MFB are observed at the periphery of the fibrotic area, suggesting existence of transitioning hepatocytes. Several reports suggest that type 2 EMT plays an important role in chronic liver inflammation. Hepatocytes from cirrhotic livers have been shown to express higher vimentin in response to TGF-β, and stronger anti-apoptosis effects compared with those from normal livers. This evidence suggests the existence of cells with features suggesting EMT, and even cells with fully completed EMT, during chronic liver injury. In short, chronic inflammation promotes pathologic type 2 EMT in the liver, and imparts mesenchymal features to hepatocytes.

Type 3 EMT is observed during the HCC progression process. Ras, WNT, β-catenin, and TGF-β have been shown to enhance the malignant features of HCC [125]. Among them, Ras-MAPK increases Snail and Slug expression, and promotes EMT by down-regulating E-cadherin [126,127]. Snail significantly increases with HCC development, accelerating cancer invasion. The down-regulation of E-cadherin has been shown to play an important role in the acquisition of metastatic capability by HCC.

Increasing evidence suggests that chronic TGF-β stimulation promotes EMT as well as cancer stem cell properties and a higher invasive capability in HCC [128,129]. CD44-positive HCC cells have been reported to exhibit the features of EMT and show increased potential for chemoresistance [130]. Smad3 has been reported to promote tumor progression at advanced stages by inducing EMT and enhancing pro-metastatic transcription factors such as Snail and Slug [131,132,133,134]. Recently, it was also reported that Golgi protein 73 (GP73) promotes EMT and invasion by Smad2 activation in HCC cells [135]. Moreover, G protein-coupled receptors and their ligands can transmit signals through linker and the C-terminal phosphorylation of Smad2/3 [136]. Down-regulation of Smad7 expression has been shown to promote HCC metastatic potential by facilitating EMT [137]. Interestingly, the recovery of Smad3 signaling can restore the chemosensitivity of HCC cells [138]. Recently, several kinds of microRNAs (miRNA) have been reported to regulate Smad/EMT signaling; in contrast, miRNA-125b and miRNA-708 suppress Smad2 and Smad3 pathway and attenuate EMT [139,140], miRNA-520g down-regulates Smad7 and promotes EMT in HCC cells [141]. These data suggest that TGF-β/Smad-induced EMT signaling have important roles in the generation of highly invasive and chemoresistant cells with stem cell-like features in HCC. 

## 10. Conclusions

TGF-β was initially characterized in terms of its ability to induce malignant behavior in mesenchymal cells such as HSC, and therefore was designated a “transforming” growth factor. Together with growth factors that activate the receptor tyrosine kinase/Ras pathway, TGF-β stimulated the proliferation of fibroblasts under anchorage-deficient conditions, which is a hallmark of cellular transformation. Several years later, TGF-β was shown to be a strong growth suppressor in normal epithelial cells such as hepatocytes after transient Ras activation. During the transition from human benign tumors to carcinomas in situ, tumors with Ras-activating mutations, TGF-β gradually loses growth inhibitory effects. However, Ras and TGF-β signaling work in concert to promote cancer cells to undergo EMT in cells at the invasive fronts of human cancers, where they soon acquire invasive and metastatic phenotypes. Insights into stepwise human carcinogenesis have emerged from recent detailed analyses of cell type-specific and context-dependent TGF-β signaling processes directed by multiple phospho-isoforms of Smad mediators. 

To elucidate the involvement of TGF-β signaling in a pathophysiologic condition, we need to understand how this signaling interacts with the disease as well as with established and potential therapies [11]. For example, thrombin promotes PAI-1 expression by up-regulating the Smad2L pathway in keratinocytes [142]. Flavopiridol has been reported to inhibit pSmad2L/C and prevent atherosclerosis [143]. Unraveling the molecular mechanisms involved in the progression to HCC is of fundamental importance in guiding the development of effective prevention and treatment for HCC. Here, we have sought to offer an overview of the recently reported clinical and basic research concerning fibrocarcinogenesis in the liver. In this review, we highlighted the reversibility and sometimes irreversibility of Smad phospho-isoform signaling favoring tumor suppression or fibrocarcinogenesis in hepatitis virus-related liver diseases. In addition to its pathogenic importance, Smad phospho-isoform signaling might be useful as a new biomarker to predict likely success of pharmacologic interventions intended to suppress human heptatic fibrocarcinogenesis.
